# Why and when successful rural malaria control became a local problem – Palestine 1922

**DOI:** 10.5281/zenodo.17512696

**Published:** 2025-11-01

**Authors:** Anton Alexander

**Affiliations:** 1 BC Business Centrum, Elscot House, Arcadia Avenue, London N3 2JU, United Kingdom.

## Abstract

Recent events, i.e., the demise of USAID, recognition of a State of Palestine, and the massacre of 7th October 2023 by Hamas, have seemingly come together to remind of the obstacles the Zionists had to overcome in Palestine more than 100 years ago when launching the start of the first sustainable rural malaria control programme. Here, I examine how the Zionists dealt with situations of a similar nature to these events all that time ago.

## Background

**Figure 1.**
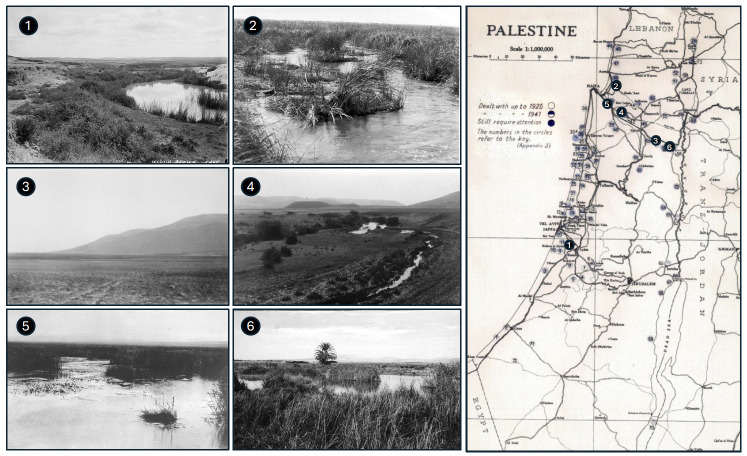
The Palestine Department of Health map of the 74 more important swamp areas in the early 1920s [1]. Examples of areas desolate due to malaria at that time: 1. Wadi Rubin; 2. Swamps near Kurdani, south of Acre; 3. Mount Gilboa, Jezreel Valley; 4. Swamps near Kfar Hassidim; 5. Swamps near Kishon; 6. Nir David, Beth Shean Valley. Images: Courtesy of the Palestine Exploration Fund Archive, London and the Central Zionist Archives, Jerusalem.

Figure 1 shows examples of conditions prevailing in various locations in Palestine over 100 years ago. Already in the 19th century, Palestine was known to be notoriously malarious, rendering much of the country desolate, almost empty, either uninhabitable or sparsely populated.

There, in the final year of WW1, from 1 October 1918 onwards, over 20,000 men, that constituted over half the British Army, were to die or collapse from malaria after they had been exposed to the disease for only 11 days immediately after 19 September. Dr. Manson-Bahr, a medical officer in the British Army in Palestine and a future director of the London School of Hygiene and Tropical Medicine, described Palestine in 1919 as one of the most malarious countries in the world.

After the defeat of the Turkish Army in 1918, a Palestine Mandate, on behalf of the League of Nations, was assigned to the United Kingdom, and operated from 1920 to 1948 by a British civil administration.

In 1920, Dr. Israel Kligler, an American public health scientist, and an idealistic Zionist Jew, arrived to settle in Palestine, with a view to address the malaria problem, which was to be dealt with as a priority. Failure to deal with malaria would have meant the Zionist dream of settlement of Jews, of a Jewish Homeland, in Palestine would have been unlikely, if not impossible.

Basically, Kligler was without significant funding and without the manpower to deal with malaria in the manner seen from 1904 onwards during the Panama Canal construction and, for 6 months before 19 September 1918, in Palestine. He therefore devised a fresh approach for malaria control but with a view to also achieving sustainable malaria control. The results of his initial demonstration, although being a very modest pilot project, were impressive enough for significant funding to be made available for malaria control on a countrywide scale.

## Recent Events

### USAID cuts

The United States has recently cut off its financial aid for many projects around the world, including those involved in the fight against malaria. Until Kligler’s arrival in Palestine, previous attempts at controlling malaria, namely during the Panama Canal construction (1904-1913) and in Palestine by the British Army in 1918, had been conducted only on a large scale and both operations had been very costly. Neither attempt had considered or intended sustainable control as an outcome.

The intention of control at the Panama Canal had been only to enable construction of the canal. The 1918 British Army Palestine control had lasted for six months and was only intended to protect the British Army from malaria whilst preparing for its final victorious battle against the Turkish Army.

Kligler was a Zionist and his intention was also to control malaria – but his intention was also to do so sustainably in order to make Palestine habitable - and to thereby possibly enable a Jewish homeland.

Malaria was an obstacle in all three cases obstructing the respective ultimate goals. Therefore, malaria control had become a priority as otherwise the ultimate goals were unachievable. Whilst the 1904 Panama Canal and 1918 British Army situations came with funds and manpower, Kligler was without both the funds and manpower to conduct anything resembling these previous large-scale achievements.

Kligler would have read of the methods for large-scale malaria control employed at the Panama Canal and also by the British Army in Palestine. Those methods had employed thousands of men and had been executed at enormous expense. Kligler had none of these resources and therefore realised he had to view his task by employing a different approach.

### Recognition of a Palestinian state

A spotlight is being shone on the meaning of, or reference to, ‘Palestinian’ and which is beginning to lend to confusion. Is the reference meant as an alternative to ‘Arab’ which perhaps explains why some Israeli Arabs may describe themselves today as Palestinian Israelis? Or does it refer to an Arab originally from Mandate Palestine before 1948? Or is it anyone, not just an Arab, who had been living in Palestine before 1948? The description ‘Palestinian’ appears to have evolved over the last 60–70 years. It seems a muddle which has grown and been compounded by the United Nations with its treatment of the 1948 refugees from the Arab-Israeli war by uniquely suggesting a refugee status can be inherited by the next generations! Accordingly, references to a ‘Palestinian’ can now also, it seems, be a reference to refugees, regardless of generation, regardless of ethnicity. It is confusing and a muddle.

Kligler also had to deal with a similar muddle in the 1920s and which, as will be seen later, created a problem for him, although it was then more of a cultural/ethnical muddle. A 1922 Palestine Government Census [2] had revealed a total of only 389,534 rural/village inhabitants for the whole of the country. These rural inhabitants, in fact, represented the majority of a total Palestine population of only 757,182 (103,331 in Bedouin/tribal areas and 264,317 in municipal/ town areas - Jews were mainly part of the municipal/ town statistics.)

In particular, the Census had also included statistics showing ‘Languages habitually spoken in Palestine’ and listed 40 different languages! The Census classified inhabitants only according to religion, not ethnicity. With so many languages indicating so many different backgrounds and cultures and yet, with so few inhabitants, it served to confirm the notion then that a single Palestinian identity or people would have been unthinkable. The only thing they all had in common was that they were all in Palestine at the same time during the Census. They were Palestinian Muslims, Palestinian Jews, Palestinian Christians, etc.

Kligler set about familiarising himself with the country upon his arrival in Palestine in 1920. He had beforehand read extensively about the malarial conditions then known before his arrival, but he may not have known in great detail the backgrounds or cultures of the inhabitants (albeit few in number). However, he was likely to have discovered for himself the mix of peoples whilst travelling the country inspecting conditions.

Kligler was later to describe the diversity he encountered in his 1930 text-book [3]:

*“Palestine is a country of mixed peoples, many religions, and all gradations of civilisation. There are Jews from the Orient and Occident, and side by side there are city Arabs, the peasants, and the roving Bedouins. There are Moslems, Jews, and Christians, people of every sect and denomination. And there are as well all gradations of culture, from the Nomadic, pastoral Bedouins to the most modern industrialised groups. A more heterogeneous population can hardly be found anywhere in the world.”*

There was no national identity and no cohesion between the inhabitants other than to their own religion, individual group, or tribe. There was no notion of a Palestinian entity or nation. Palestine was made up of mixed peoples who happened to be in Palestine at that moment.

An explanation for some of the mix was that, before WWI and as the Ottoman Empire contracted, several Muslim communities were expelled from those other parts breaking away from the Ottoman Empire and seeking their independence. These expelled communities included Circassians (i.e. probably 1.5–2 million Muslim refugees from the Caucasus, Russia), Algerians, and Bosnians, and were periodically ‘resettled’ by the Ottoman Empire into the region, no matter how desolate the region.

By way of an interesting illustration for this paper of such ‘resettlement’, Amman (now the capital of the Kingdom of Jordan) was, prior to 1878, comprised only of abandoned Roman-era ruins. In 1878, the Ottoman authorities with a view to reinhabiting the area and ultimately reversing the desolation, introduced the first group of Circassion exiles to the ruins of Amman who were later joined by further Circassions groups as directed by the Ottoman authorities. Therefore, today’s Amman, Jordan's primary city, probably the largest city in the Levant region and fifth-largest city in the Arab world, was in fact begun by Russian Muslims – not by Arabs.

Some refugees from these expelled Muslim communities were also sent to Palestine.

The Palestine Exploration Fund (patron Queen Victoria) had initiated a full survey of Palestine from 1872 onwards and from July 1872, the survey was carried out by two British Army engineers. An example of reference to Algerians in Palestine occurred as a result of an incident in July 1875. During the survey, the two Army engineers were attacked near Safed in the north of Palestine and were injured so badly, the survey was suspended for 15 months during which time they both returned to England to recover. Subsequently, in September 1876, the attackers of the engineers were put on trial in Palestine. Prison sentences were handed out to 8 described as ‘natives of Safed’ for withholding evidence and to the other 8 described as ‘Algerians settled in Safed’ for the actual attack.

Kligler commented [3] in 1928 of his early experience in Palestine concerning such exiles:

*“One passes a Circassian village half encircled by marshes – one of the outposts colonized by the Turks forty years ago with hardy stock from Circassia, nine hundred strong. Now there are scarcely fifty souls left in the village, mostly native stock and all stamped with the effects of malaria. This condition is repeated at Ramadan, Jelile, at Nebi Rubin, in short, all along the coast.”*

Whilst all the expelled communities that had been resettled by the Ottomans in Palestine would have been Muslim, it is unknown what proportion of those inhabitants that had been included in the Census was made up of these expelled Muslims. Further, whilst such expelled communities were Muslim, many were not Arabs, and which can possibly explain why the census found it more convenient to make a classification based on religion rather than on ethnicity.

### Hamas and the 7 October massacre

Hamas is a branch of the anti-semitic Muslim Brotherhood, and the barbarity of the pogrom in Israel of 7th October 2023 demonstrated its hatred of Jews.

Kligler was confronted with a similar situation. In 1922, Haj Amin el Husseini, a member of an influential Arab family in Palestine, had been appointed the Grand Mufti of Jerusalem, becoming the leader of the Palestine Arabs. The Mufti’s dislike of all Jews (not just of Zionists) was well known, having already been implicated in a massacre of Jews in Jerusalem in 1920.

1922 to 1929 were years of peace. By 1926, according to a 1937 Palestine Royal Commission report, the British Government had felt able to reduce the forces available for maintaining order to a very low strength because “For some time past, Palestine has been the most peaceful country of any in the Middle East”.

But in August 1929, a massacre of Jews by Arabs took place in a number of towns in Palestine. The subsequent Government Commission enquiring into these 1929 disturbances implicated the Grand Mufti. There followed further violence during the 1930s, which began to be directed not only against the Jews but also against the British authorities. Even staff engaged in anti-malaria work became a target for Arab violence.

**Figure 2.**
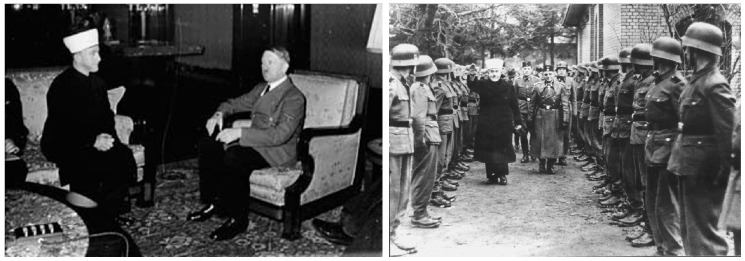
During World War 2. Left: The Mufti with Hitler in Berlin; Right: Inspecting Nazi soldiers.

The British recognised the Mufti’s role in the disturbances not only against the Jews but then also against the British, and accordingly, just before the outbreak of WWII, the Mufti fled Palestine and eventually spent WWII with Hitler in Germany assisting with Nazi war efforts (Figure 2).

Before and after the Mufti had fled, there were attacks by Arabs who supported the Mufti on those Arabs who didn’t, causing the latter to flee the country in fear for their lives. This was at a time when there were many Jews desperate to enter Palestine who had managed to escape from persecution in Nazi Germany. The intensity of the violence of Arab against Arab had in 1938 caused the number of Arab refugees fleeing the Mufti’s supporters to exceed the number of Jews arriving in Palestine who had fled from Nazi Germany.

After WWII, the Mufti managed to escape and lived in exile in Egypt and Lebanon where he continued to propagate anti-Jewish propaganda until his death in Beirut in 1974. Despite his Nazi collaboration during the war, he evaded trial at Nuremberg for war crimes and continued his nationalist activities, opposing the establishment of the State of Israel.

The Mufti was one of the last of the fascist leaders to have been with Hitler during WWII and to survive the war. From the late 1920s onwards, the Mufti had sown the seeds and influenced many Palestine inhabitants with the same hatred that had existed in Nazi Germany in the 1930s. That hatred ultimately had led to death and destruction in Nazi Germany and which same hatred sown by the Mufti appears to have travelled down time and was now sadly seen in Gaza on 7th October 2023.

The fact that police were used to guard and protect anti-malaria workers from attacks by those sympathetic to the Mufti’s views not only hampered the work but also demonstrated indifference to the wellbeing of the community at large.

## Kligler’s New Approach

As explained above, Kligler had to contend with situations similar to events having taken place recently. Fortunately for Kligler, he was initially provided with funds by a private Zionist supporter, but whilst these funds were limited, they were still sufficient to enable him to conduct modest experiments in methods of malaria control in three or four highly infected small communities/sections of the country. No radical drainage was attempted. The primary objects of the experiments were to ascertain (1) whether malaria control was possible with limited means and (2) the cost of such control. But while the eye was constantly kept on the cost, the experiments were so planned that exact data on the various items associated with malaria control could be collected and tabulated.

Screening, mosquito nets, and other measures of personal protection were not attempted because the cost was prohibitive. In order to check and control comparable results, these experiments/demonstrations had to all be conducted at the same time. The areas selected differed from one another in many essentials, but were sufficiently alike to enable Kligler to compare and check results. On the one hand, each place was distinctive though typical of other places in Palestine, but on the other hand, the character of the population and the problems of mosquito control were sufficiently alike to make it possible to compare costs, etc.

It was here that Kligler was obliged to take note of the mix within the population, of the great diversity in the inhabitants. As mentioned, because he needed the character of the population in each place/demonstration area to be sufficiently alike to make possible comparisons of results, rather than choose 4 settlements at random that could possibly complicate comparisons, he opted for 4 settlements with fixed Jewish populations with one or two of them having floating Arab populations. Average populations for the 4 settlements ranged between 100 to 440 including floating populations.

Just to remind, no radical drainage was attempted. His experiments principally included an anti-mosquito campaign which was aimed principally at the larvae and included a thorough survey of the area, with the emptying of all water-containing receptacles, the covering of cisterns, water-barrels, and the spraying of pools and other bodies of stagnant water with petroleum at regular intervals. It also included such measures as drying of certain swamps, the repair of irrigation ditches, and the institution of intermittent irrigation which were all carried out with surprisingly good effects. Education of the inhabitants in these sections was also particularly stressed.

The results were sufficiently positive to justify the preparation of a country-wide scheme with a proportionately larger budget. He had results that he could show to prospective funders instead of going, cap in hand, with merely good ideas. The funders understood the goal, namely to make Palestine habitable, and they agreed this as a priority with Kligler.

Kligler had come to the funders with a completely new approach and he wrote [3]:

*“We have come to realise that malaria is eminently a local problem, and that a successful attack is possible only after a careful study of the local conditions combined with systematic experiments with the method or methods most likely to give the desired results.”*

(Such a comment is so far removed from today’s malaria methods which usually are applied over extensive areas and which include differing backgrounds, localities and situations. Such areas include many variations all grouped together. It is unclear how reliable meaningful data may be collected together over such extensive areas.)

Immediately after WWI, the British Mandate had neither the funds nor the interest in controlling malaria in rural Palestine. Therefore, based on the results that Kligler provided, the American Zionists (the Joint Distribution Committee) agreed to fund a complete anti-malaria organisation, the Malaria Research Unit (MRU), for over four years to be attached to the Palestine Mandate Health Department. The malaria control was directed initially around the Jewish settlements and neighbouring Arab settlements but with a view to slowly cover the entire country. With the funding available, Kligler could then move forward to implement a country-wide scheme.

As manpower was also necessary, Kligler required the assistance of local inhabitants and so he had to interest them in the work. He wrote [3]:

*“The education of the inhabitants was … by no means the least important element which conditioned the success of the work. Without active cooperation on the part of the people, the work would have been only partially successful. It was possible to obtain their active co-operation only after they understood fully the significance and value of the work.”*

This was an important comment as each locality provided its own local conditions, often different in some aspects from a neighbouring locality. This is in contrast to many of today’s malaria methods conducted over extensive areas, today’s methods often tending to only lend themselves to a very general education without providing a solid practical education and guidance for affected communities. Kligler’s method of dealing with malaria as a local problem enabled the education to be focused on a smaller area/scale and with the inhabitants considering specific problems as against a general education. Kligler’s inhabitants were therefore more likely to have been of practical use with attention being given to local problems with local solutions.

Kligler’s new funding was intended, this time, to include cost of drainage works, or at least contributions to the cost, as explained in the following extract [4] from the 1923 Annual Report of the MRU for the Palestine Department of Health. It explained how the inhabitants were moved to attend the demonstrations:

*“Another phase of the activity of the [Unit] was the permanent elimination of breeding places. … we attempted to stimulate co-operative drainage undertakings by the villages and settlements. We offered to contribute part of the cost of the work on condition that the inhabitants of the communities affected contribute the rest either in money or in labour. This procedure helped to awaken the interest of the inhabitants in their malaria problem and has already borne fruit in several completed and projected drainage schemes. This year’s experience demonstrated that many of the important swamps now existing in the Demonstration Areas can be eliminated at a relatively small cost, and that small grants given to those settlements ready to bear a part of the cost will help them to remove permanently at least some of the chief sources of malaria.”*

Each village or settlement was free to join but, as pointed out, if it did join, it was obliged to contribute towards drainage and other works. It also presumably provided the inhabitants with a sense of ownership of the work and therefore a greater interest in maintenance of such works.

The diversity of cultures and backgrounds (and sometimes even of languages) of the inhabitants was a hindrance for Kligler in that a successful approach to teaching or educating one community may have been completely different for a neighbouring community or settlement with a different culture, background etc., and the process may have had to begin again of establishing a level at which to begin the education.

After the first four-plus years, the Mandate recognised the successful control method and agreed to begin to assume financial responsibility but with the American Zionists contributing decreasing amounts each year.

### Consequences of effective education

As a result of the education, the co-operation of the inhabitants overall was effective and remained strong over the years despite the intimidation by the Mufti with attempts to disturb. Indeed, the very fact that the control was so successful was testimony to the education, the strength of the cooperation and its ability to withstand attacks by the Mufti.

In 1925, the League of Nations Malaria Commission had heard of the successful anti-malaria works underway in Palestine, and came to inspect. After the Commission had inspected the anti-malaria works, a comment in the subsequent 1925 Malaria Commission Report was of very great significance and relevance:

*“Above all, it has succeeded in inducing the people of the country to take an interest in health problems and to co-operate in measures for the prevention of disease.”* [5]

Without Kligler’s education of the inhabitants, the 1925 Report wouldn’t have been able to make such a claim. British Government Commissions in 1937 and 1938 reported an astonishing and abnormally high – possibly unprecedented – rate of natural increase in the non-Jewish population due to a lower death rate brought about by the anti-malaria campaign. Thus Palestine, a previously almost empty desolate country, was to experience an ‘abnormally high rate of natural increase’ and ‘astonishing change in the population’ which was confirmed in the Statistical Year Book of the League of Nations 1931/32 as the highest in the world [6].

Thanks to sustained malaria control, the pre-WWI Palestine bore hardly any similarity or resemblance to the post-WWI Palestine, not only in relation to the extent of reclaimed and available usable and habitable land but also in relation to the number of inhabitants. Between 1922-1948 (26 years), the number of Muslims/Christians almost doubled, the bulk of the increase being made up by natural increase, whereas the increase in Jewish numbers was due mainly to immigration. Based on the United Nations figures, interestingly, the great increase in numbers of both Jews (524,536) and Muslims/Christians (563,842) during the period 1918-1947 was almost the same for both communities. Almost all historical narratives fail to explain why, from 1918, from the end of WWI onwards, there were later so many more Muslims and Christians in 1948 at the time of the creation of the State of Israel than there were in 1918. Very few appear to notice, and even fewer ask why.

## Conclusions

Kligler’s scientific method may also perhaps be considered a marketing exercise. He presented a modest pilot project that did what the funders wanted. Kligler was a Zionist, as were the funders, so Kligler already knew what was required. Nevertheless, the funders were still not prepared to provide funds unless and until real solid evidence could be provided of the likelihood of success. Kligler’s pilot project did that.

To be effective today, malaria control as a package would probably have to be ‘sold’ as a tool that can clear away the obstacle, namely malaria, that had prevented an ultimate goal from being achieved. Sadly it appears in this day and age, mere good health as an ultimate goal is often not attractive enough for many governments. Therefore as a suggestion, any control package could include perhaps also some economic benefit or advantage that could be achieved by controlling or eliminating the disease. But that benefit or advantage would probably have to be prepared and demonstrated separately as a serious economic project in its own right by economic experts.

It should not be forgotten, Kligler had to ‘sell’ the control project to both the funders and also to the inhabitants. They both, funders and inhabitants, had to see a benefit, otherwise failure would have been likely.

Finally, it should be noted Kligler did not require huge amounts of funding immediately, but only as and when needed. Kligler’s approach enabled inhabitants/communities to join the control program in their own time or even not at all, (although presumably there could ultimately come a time where isolated pockets of malaria may threaten the controlled areas if left untreated). But certainly it was a gentler way of dealing with matters as inhabitants would only have been requested to join once they had seen successful control elsewhere in the country.
